# ECM-Preserving Decellularization of Dermis via Subtle Cell Membrane Disruption Induced by Subcritical Dimethyl Ether

**DOI:** 10.1021/acsomega.5c05071

**Published:** 2025-08-21

**Authors:** Naoko Nakamura, Kota Tanabe, Hiroko Fukuhara, Kohei Otsuki, Masatoshi Shimotomai, Shunya Shiba, Tsuyoshi Kimura, Akio Kishida

**Affiliations:** † College of Systems Engineering and Science, Shibaura Institute of Technology, 307 Fukasaku, Minuma-ku, Saitama-shi, Saitama 337-8570, Japan; ‡ Graduate School of Engineering and Science, Shibaura Institute of Technology, 307 Fukasaku, Minuma-ku, Saitama-shi, Saitama 337-8570, Japan; § Department of Biomedical Engineering, Faculty of Life Science, 13117 Toyo University, 48-1 Oka, Asaka-shi, Saitama 351-8510, Japan; ∥ Institute of Integrated Research, Institute of Science Tokyo, 2-3-10 Kanda-Surugadai, Chiyoda-ku, Tokyo 101-0062, Japan

## Abstract

Decellularized tissues are used as biomaterials for transplantation. Many decellularized tissues in clinical applications are prepared using surfactants; however, we have developed a new decellularization method that uses subcritical dimethyl ether (DME) instead of surfactants. Subcritical DME perfusion is usually used for lipid extraction; therefore, by perfusing tissues with subcritical DME, phospholipid cell membranes may be destroyed. DME vaporizes at room temperature and pressure, therefore, it is expected that it will not remain in the decellularized tissues and will not be toxic. In this study, subcritical DME was perfused into the porcine dermis, and the sample was subjected to DNA degradation to produce a subcritical DME-decellularized dermis. The subcritical DME-decellularized dermis showed good cell response in vitro and in vivo. In addition, we investigated the mechanism of the subcritical DME decellularization method and found that surfactants dissolve the entire cell and almost remove it; however, subcritical DME causes minor damage to the cell membrane and removes the cell nucleus through DNase treatment while leaving some of the cell membrane intact. These results suggest that subcritical DME-decellularized dermis is nontoxic and has the potential to develop highly functional decellularized tissues, such as extracellular vesicles, unlike decellularized dermis prepared with surfactants.

## Graphical abstract

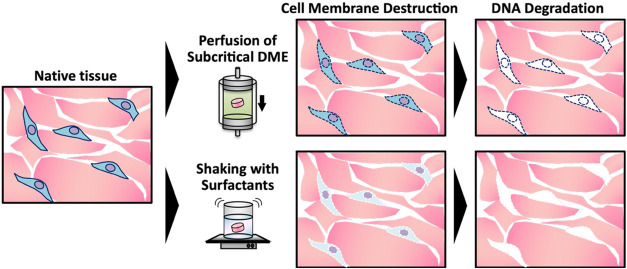

## Introduction

Decellularized tissue consists of an extracellular matrix (ECM), from which the cytoplasm and cell nuclei have been removed from living tissue, and has been used as a scaffold for tissue repair and reconstruction.^1–3^ In 1999, CryoLife Inc. was the first to use decellularized porcine aortic valves in clinical research. Currently, various decellularized tissues, such as the skin and pericardium, are commercialized and used in clinical applications. Decellularized tissue is not subject to immune rejection and expected to lead to tissue regeneration in patient cells. However, the commercialization of decellularized tissues with complex three-dimensional structures is still limited, and research is ongoing.

There are various decellularization methods that can be broadly classified into three categories: physical, chemical, and biological.^4^ Decellularization cannot be adequately achieved using only one of these methods, therefore, a combination of several methods is generally used. Although decellularization treatments aim to remove only cellular components while maintaining the three-dimensional structure and functionality of the ECM of biological tissues, it is difficult to do so without damaging the ECM, regardless of the method used. Therefore, it is necessary to select a decellularization method that is appropriate for each tissue and minimizes destruction of the ECM.

Chemical processing is the most common method for removing cells from living tissues. Surfactants and other agents are used to break down the lipid bilayer, which constitutes the cell membrane and dissolves cells in biological tissues. After this process, the DNA is degraded with DNase, and fragmented DNA is washed out of the tissue to prepare the decellularized tissue. The method using surfactants are clinically used. However, because surfactants also bind to proteins, there is a concern that they may remain in the decellularized tissue and adversely affect the surrounding cells after transplantation. Therefore, various attempts have been made to reduce the concentration of the surfactant^5^ and combine multiple surfactants.^6,7^

As an alternative chemical treatment to surfactants, there are reports of dimethyl ether (DME) being applied to decellularization; DME is the smallest ether compound and liquefies at −24.8 °C at ambient pressure. Subcritical DME is commonly used as a dehydrating and deoiling agent, and lipids can be extracted almost completely by applying subcritical DME to highly hydrated materials, such as microalgae.^8^ The advantage of these extracts is the absence of residual DME, which becomes a gas at normal pressure and temperature. In addition, subcritical DME can be used to extract both polar and nonpolar lipids. Therefore, lipid bilayers, which constitute cell membranes, can be extracted from wet biological samples. In a previous study, the decellularization of the aorta and cartilage by subcritical DME treatment was reported.^9–11^ In this study, subcritical DME was used instead of a surfactant to remove lipids from biological tissues, followed by the degradation and removal of DNA using a DNase solution. Although there have been reports on the preparation of decellularized tissues with subcritical DME, the mechanism by which subcritical DME acts on the cell membrane to enable decellularization and whether subcritical DME-decellularized tissues are biocompatible materials are unclear.

Decellularized dermis is used as artificial skin that compensates for skin defects.^12,13^ The dermis is rich in ECM components, such as collagen, therefore, it is also used to replace tissue defects in completely different areas^14–16^ and prepare scaffolds for transplanting other tissues,^17^ making it a versatile material. The degree of damage to the ECM, such as destruction of higher-order structures and denaturation of collagen, may play a role in the in vivo biocompatibility and tissue reconstruction of decellularized tissues, therefore, there is a need to balance the removal of cellular components with the retention of the ECM in an intact state. In this study, we evaluated the effects of subcritical DME treatment on the constituents and mechanical properties of the ECM, as well as on the in vivo biological response. To clarify the effects of subcritical DME on cell membranes, changes in the cell membranes composed of lipid bilayers were examined in vitro.

## Results and Discussion

### Preparation of Decellularized Dermis and Evaluation of Decellularization

For decellularization, the cell membrane was disrupted using subcritical DME or sodium dodecyl sulfate (SDS), a surfactant, and DNA was removed by DNase treatment. The appearance of the decellularized samples was almost identical to that of the untreated samples. hematoxylin and eosin (H&E) staining revealed residual cell nuclei and ECM structures (Figure 1a). After subcritical DME, SDS 5 h (SDS5) or SDS 24 h (SDS24) treatments, which disrupted cell membranes, many cells were present in the tissue. In contrast, after DNA degradation, the number of cell nuclei was substantially reduced in the subcritical DME or SDS24 treatment. In contrast, many cell nuclei were observed in the samples treated with DNase after 5 h of treatment.

**Figure 1:**
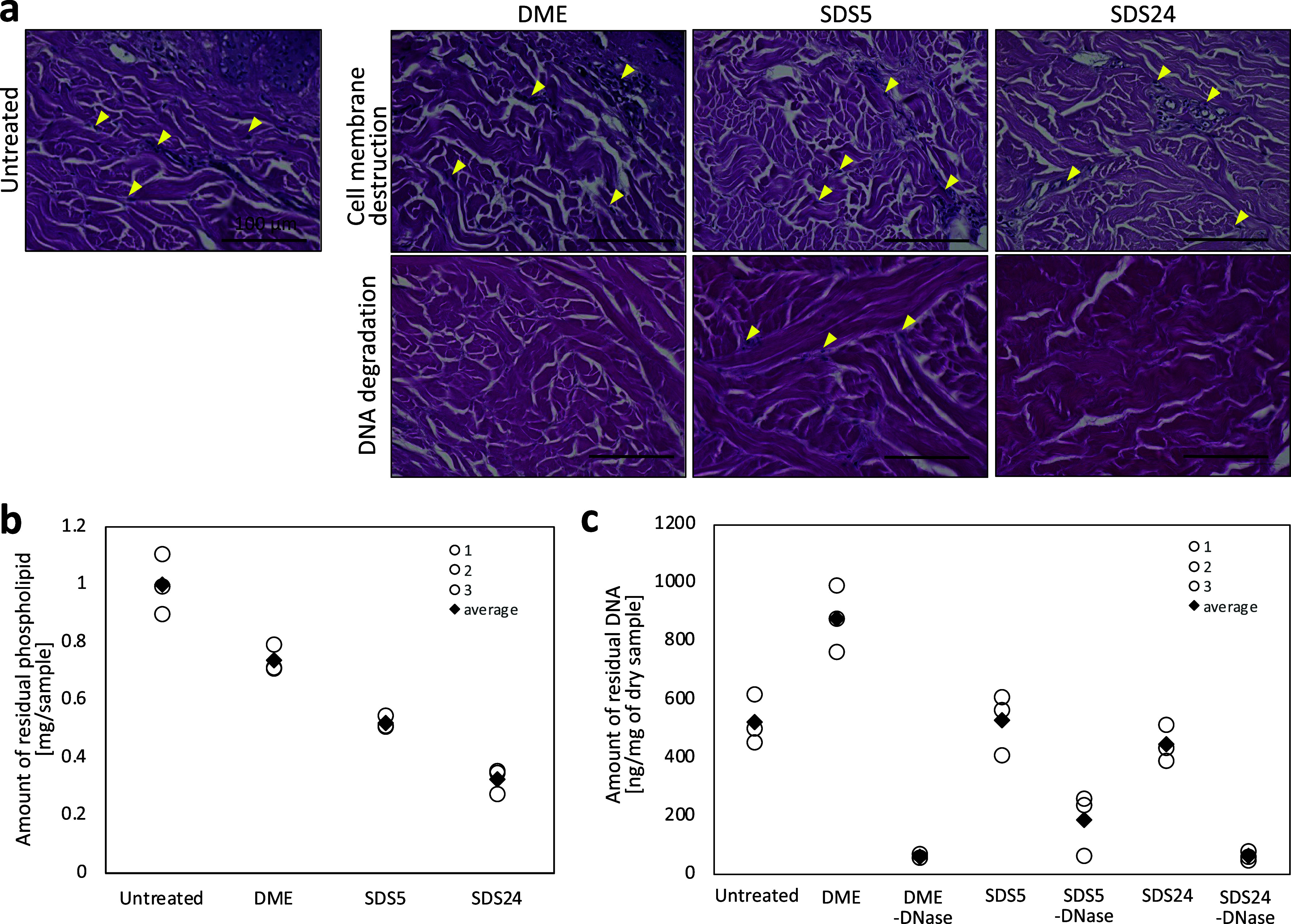
Cellular removal evaluation of decellularized dermis treated with DME and SDS. (a) Hematoxylin and eosin staining. The yellow arrows indicate cell nuclei. Scale bar: 100 μm. (b) Amount of residual phospholipids in the dermis; *n* = 3. (c) The amount of residual DNA in the dermis; *n* = 3. DME, dimethyl ether; SDS, sodium dodecyl sulfate.

The residual amount of phospholipids in the tissue was evaluated to clarify phospholipid removal using subcritical DME or surfactant treatment. The amount of phospholipids was reduced in all treatments compared to with that in untreated cells (Figure 1b). The amount of remaining phospholipids was lower in the SDS treatment group than that in the DME group. The longer the SDS treatment time, the lower was the residual phospholipid content. Although DME treatment removed phospholipids, it was less effective than the SDS treatment. Quantification of the residual DNA showed that DNase treatment, followed by subcritical DME and SDS treatments, decreased the amount of DNA in both samples (Figure 1c). Subcritical DME and SDS treatments alone resulted in DNA retention, indicating that enzymatic degradation by DNase was necessary. In addition, the amount of DNA in subcritical DME-treated samples was higher than that in untreated samples. This may be because the amount of DNA was calculated per unit mass of the sample, and subcritical DME treatment changed the mass of the sample. DME, which is widely used as a dehydrating and deoiling agent, removes water and lipids from samples. In this experiment, the samples were freeze-dried, their mass was measured, and water had no effect on the DNA content calculation. Lipids are more soluble in DME than in phospholipids.^18^ Therefore, the apparent increase in the amount of DNA per unit mass may be due to the mass reduction caused by the removal of fat using the subcritical DME treatment. Although the amount of DNA in the SDS treatment was similar to that in the untreated samples, the actual amount may be less than it appears because the surfactant may also be responsible for mass loss due to the detergent effect of fat and protein.

### ECM Structure and Mechanical Properties of the Decellularized Dermis

Elastica van Gieson (EVG) staining revealed the dermal tissue with collagen fibers in red and elastic fibers in black. The majority of the dermis is composed of collagen fibers, and elastic fibers accompanied these collagen fibers. In all treatments, both collagen and elastic fibers were similar in distribution to those in the untreated dermis (Figure 2a). Thus, histological analysis indicated that the composition and structure of the ECM were not significantly affected.

**Figure 2:**
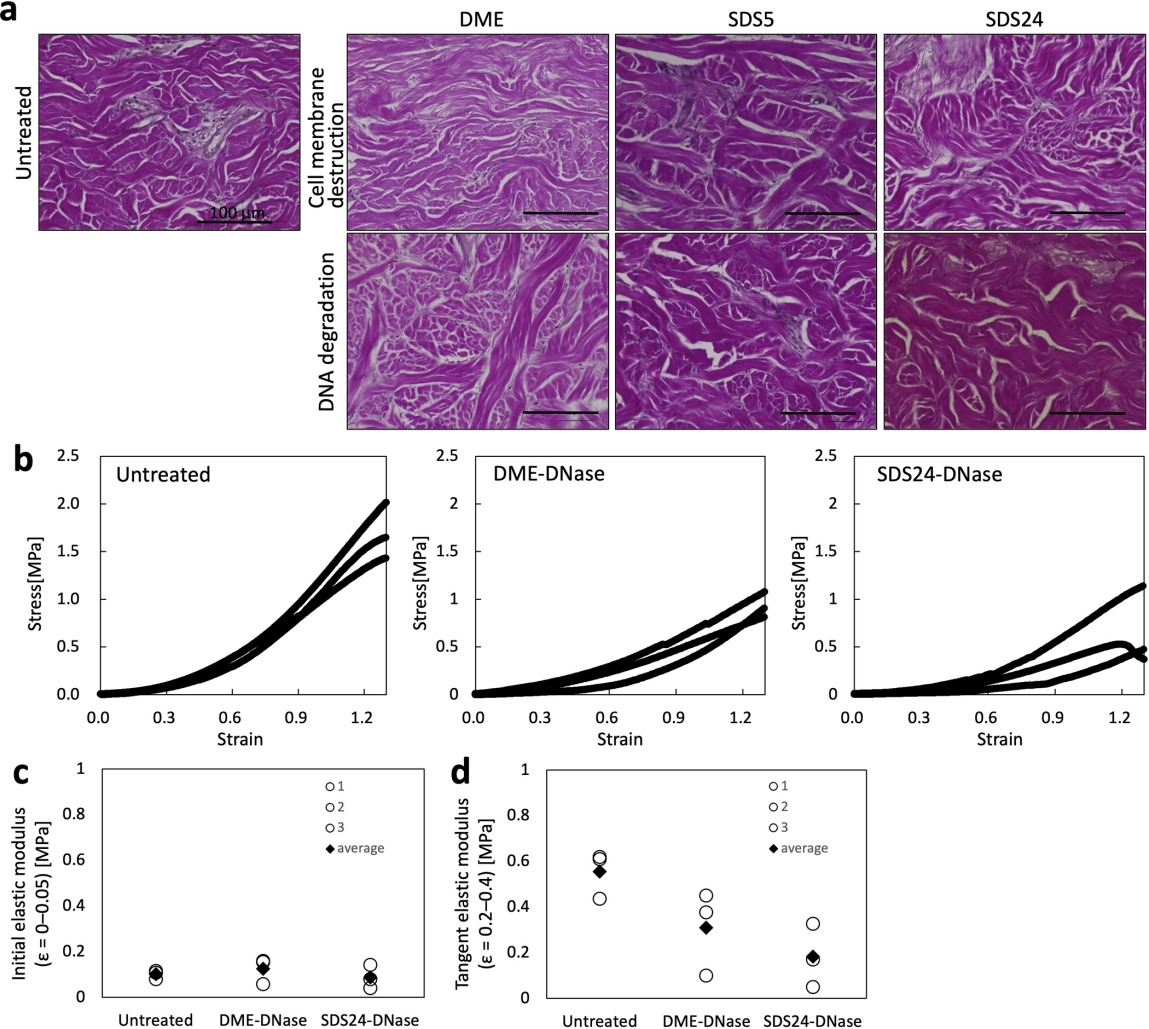
ECM components and the mechanical properties evaluation of decellularized dermis treated using DME and SDS. (a) EVG staining. Scale bar: 100 μm. (b) Stress–strain curve of the untreated dermis and decellularized dermis treated using DME-DNase and SDS24-DNase. *n* = 3. (c) Initial elastic modulus of the untreated dermis and the decellularized dermis treated with DME-DNase and SDS24-DNase, calculated from the stress–strain curve in the strain range of 0–0.05; *n* = 3. (d) Tangent elastic modulus of the untreated dermis and the decellularized dermis treated with DME-DNase and SDS24-DNase, calculated from the stress–strain curve in the strain range of 0.2–0.4; *n* = 3. DME, dimethyl ether; EVG, Elastica van Gieson; SDS, sodium dodecyl sulfate.

Mechanical testing revealed that the stress response to strain (ε) decreased in both DME-DNase and SDS24-DNase compared to the untreated dermis (Figure 2b). The initial elastic modulus (ε = 0–0.05) appeared to be similar among the untreated, DME-DNase, and SDS24-DNase groups (Figure 2c). In contrast, the tangent elastic modulus (ε = 0.2–0.4) tended to be lower in the DME-DNase and SDS24-DNase groups than in the untreated group (Figure 2d). These results suggest that DME and SDS treatments may soften the tissue under moderate strain. There was little difference between the DME-DNase and SDS24-DNase treatments.

### Investigation of Decellularization Mechanism of Subcritical DME Treatment

To elucidate the mechanism of decellularization by subcritical DME and SDS treatments, the effects of subcritical DME and SDS treatments on cell membrane disruption and nucleation were evaluated in cultured L929 cells. All cells were lost when treated with 1% SDS, the condition under which the dermis was decellularized, subsequent experiments were conducted with 0.02% SDS, in which cells remained after treatment. The subcritical DME treatment was performed under the same conditions as those used for decellularization. To compare phospholipid removal by organic solvents, cells were treated with ethanol. Scanning electron microscopy (SEM) observations showed that L929 cells immersed in DME and ethanol had small holes in the cell membrane; however, most of the cell membrane remained intact (Figure 3a). This result was consistent with the high phospholipid residues observed when the tissue was treated with DME (Figure 1b). However, L929 cells immersed in 0.02% SDS and 0.05% TritonX-100 showed a significant decrease in the upper cell membrane, exposing the cell interior. In Calcein-AM/PI staining, which is used to determine cell viability, the cell nuclei of living cells are not stained red because PI cannot penetrate the cell membrane of living cells. However, when cells die and the cell membrane is damaged, the cell nuclei are stained red by PI. Cells treated with subcritical DME or surfactants containing 0.02% SDS or 0.05% Triton were not stained green by Calcein-AM, which indicated survival under all conditions, and the cell nuclei were stained red by PI (Figure 3b). Thus, in all treatments, the cell membrane was damaged. However, the fluorescence intensity of the PI differed. PI intercalates and binds to the double-helix structure of DNA, the fluorescence intensity depends on the number of DNA double helices. Therefore, it is likely that subcritical DME treatment disrupts the cell membrane but has little effect on DNA. In contrast, when the cells were treated with EtOH or surfactants, the DNA was reduced. Subcritical DME treatment was less able to disrupt cell membranes than surfactants, and acted on DNA less than EtOH and surfactants. Under 1% SDS in decellularized tissue, cells were lost when cultured cells were treated, but when tissue was treated with 1% SDS, cell nuclei could not be completely removed, and DNase treatment was necessary. This may be because cultured cells are exposed to the treatment solution and washed away during solution exchange, whereas cells in the tissue are surrounded by the ECM, which prevents the cellular components from washing out.

**Figure 3:**
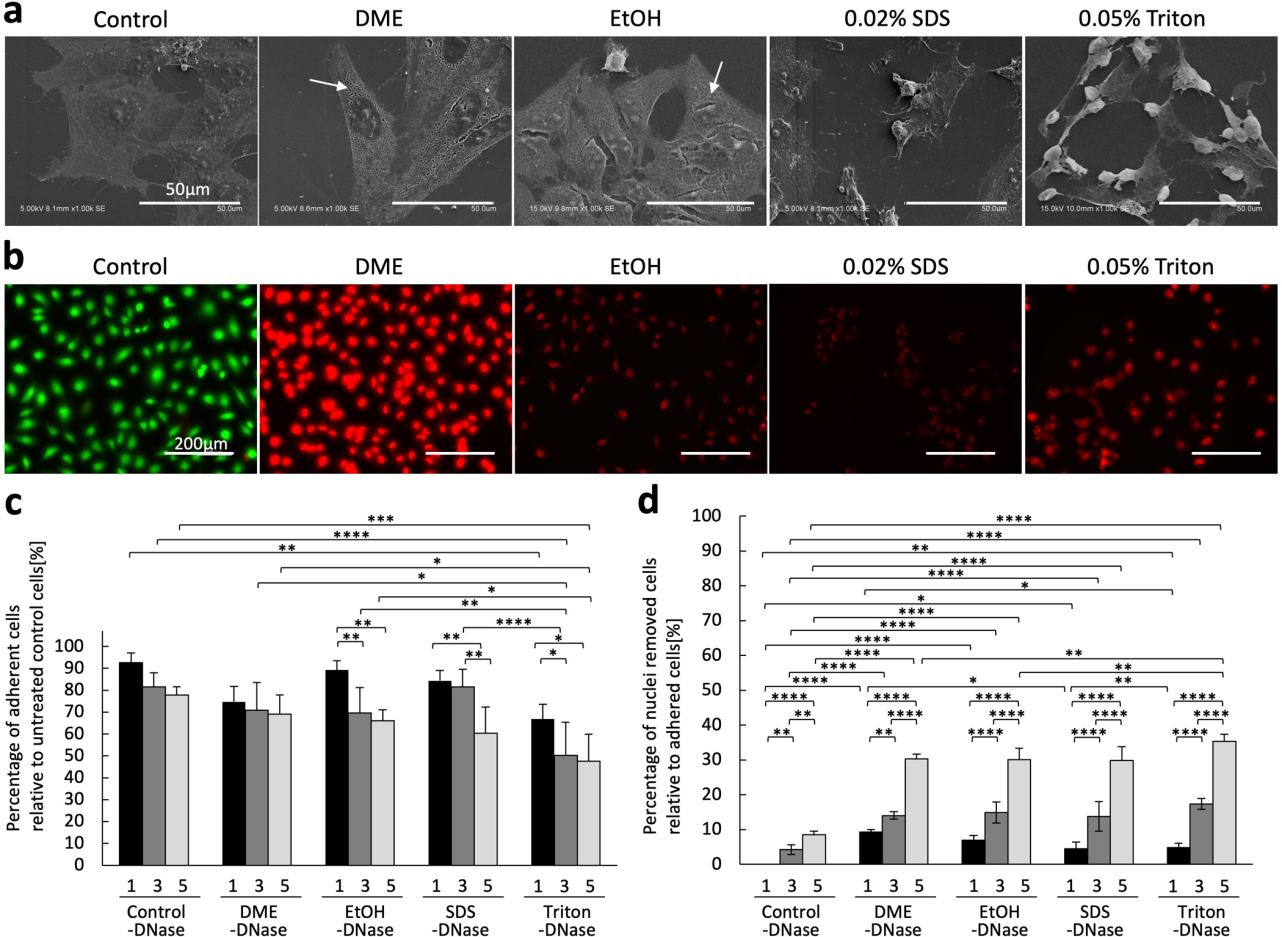
Effects of DME and surfactants on cell membranes and efficiency of cell nucleation by DNase. (a) SEM images of rat fibroblasts after DME and surfactants treatment. White arrows indicate holes or gaps created in the cell membrane by each treatment. (b) Calcein-AM/PI staining images of rat fibroblasts after DME and surfactants treatment. (c) Percentage of adherent cells relative to untreated control cells. *n* = 5. (d) Percentage of nuclei removed cells relative to adhered cells. *n* = 5. Data are represented as mean ± SD p* < 0.05, p** < 0.01, p*** < 0.001, p**** < 0.0001. DME, dimethyl ether; SDS, sodium dodecyl sulfate; SEM, scanning electron microscopy.

Cells with membranes disrupted by subcritical DME or surfactants were further treated with DNase for 5 days. The number of cells counted using phase-contrast microscopy showed that the number of cells adhering to the culture dish decreased as the DNase treatment was prolonged (Figure 3c). This was attributed to the detachment of cells from the culture dish during DNase treatment.

Merging the Calcein-AM/PI stained fluorescence image with the phase contrast microscope image revealed that in some cells, the cell nuclei were not stained with PI, indicating that the cell nuclei containing DNA were lost. The ratio of the number of cells with lost cell nuclei to the number of adherent cells was calculated, and the number of cells without cell nuclei increased as the number of days of DNase treatment increased under all conditions (Figure 3d). To clarify the effect of the treatment on cell membrane disruption and nucleation, this experiment was performed using a surfactant concentration 50 times lower than that used for tissue decellularization treatment. Whole cells were more likely to disappear when surfactants were used, even at low concentrations. However, in the removal of DNA from adherent cells, DNase treatment was equally effective in cells treated with DME as in cells treated with EtOH or the surfactant.

The changes in the amount of phospholipids (Figure 1b) and DNA (Figure 1c) in the decellularized porcine dermis (Figure 3) and cultured cells (Figure 3) indicate that surfactant treatment dissolves the cell membrane, which consists of a lipid bilayer, and washes cells, including the cell nucleus, out of the ECM. For some residual DNA, decellularization was considered to have been achieved by DNA degradation using DNase treatment. However, subcritical DME treatment alone was ineffective in removing cells, as subcritical DME treatment alone left a large amount of phospholipids and DNA in the tissue, and approximately 70% of cells in vitro remained after subcritical DME treatment alone, indicating that subcritical DME treatment itself had little effect in removing cells. However, subcritical DME treatment caused cell membrane disruption, thereby achieving DNA removal by subsequent DNase treatment. These results indicate that the subcritical DME treatment uses a different decellularization mechanism than the SDS treatment. Freeze–thawing^19,20^ and high hydrostatic pressure^21–24^ are physical decellularization methods. The mechanism of decellularization by subcritical DME treatment is similar to that of physical decellularization methods.^4^

### In Vitro Culturing of Rat Fibroblasts with the Decellularized Dermis

When the decellularized dermis and rat fibroblasts were cocultured using cell culture inserts, cells cocultured with the subcritical DME-decellularized dermis were similar in shape to the control cells (Figure 4a). The number of cells cocultured with the subcritical DME-decellularized dermis was not significantly different from that of the controls, and the subcritical DME-decellularized dermis did not affect cell survival (Figure 4b). In contrast, coculture with SDS-decellularized dermis resulted in a significant decrease in cell number. This was presumably due to the effect of SDS remaining in the SDS-decellularized dermis on the cells, as SDS has the ability to dissolve cell membranes. It is possible that this damage could be eliminated by adding an additional process to remove SDS so that no SDS remains; however, subcritical DME was found not to be as damaging to the cells as SDS.

**Figure 4:**
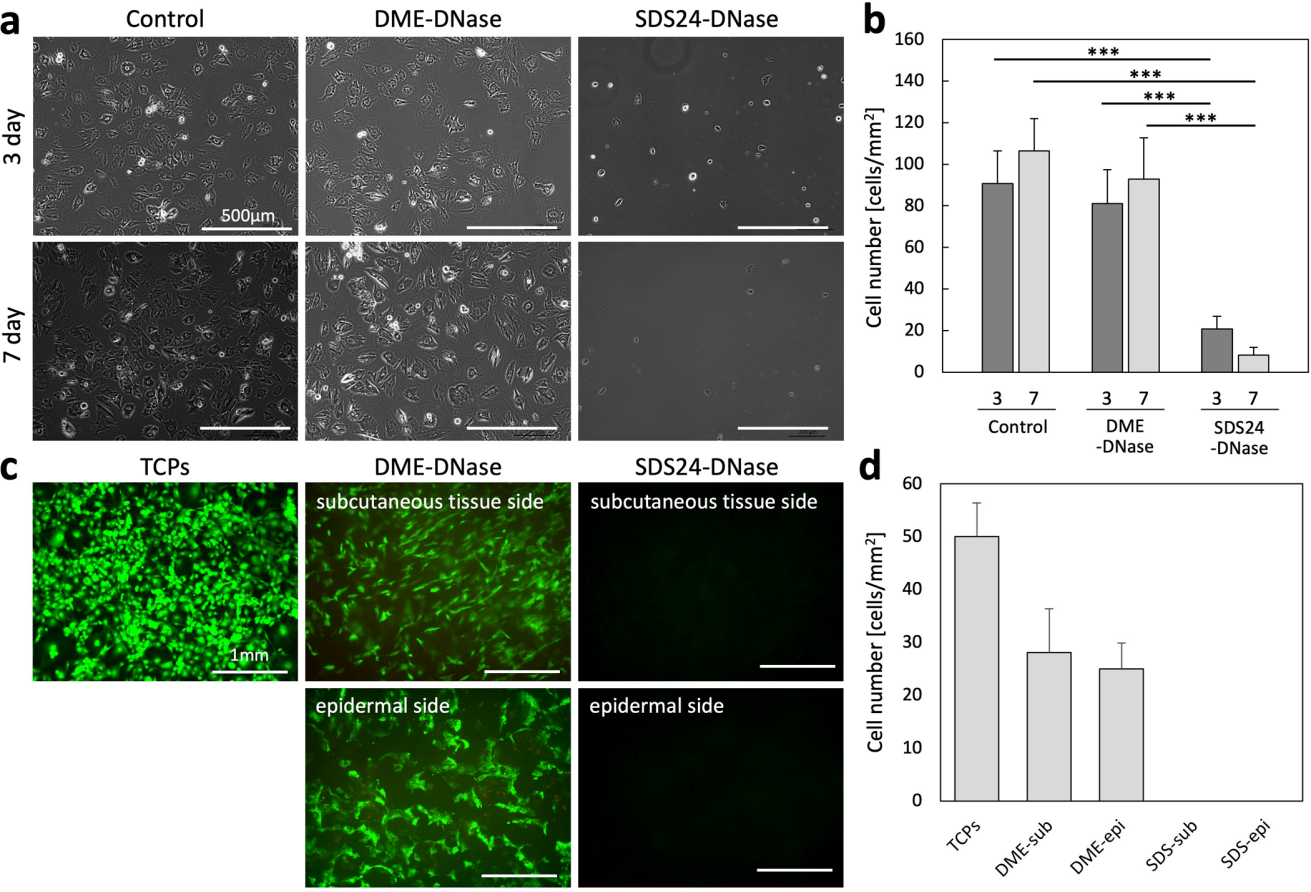
In vitro evaluation using rat primal fibroblasts. (a) Phase contrast microscope images of rat primary fibroblasts on TCPs. The decellularized dermis treated using DME-DNase and SDS24-DNase were cocultured with fibroblasts by using cell culture insert. Scale bar: 500 μm. (b) Cell number of rat primary fibroblasts cultured on TCPs for 3 and 7 days. *n* = 6, p*** < 0.001. (c) Fluorescence microscope images of rat primary fibroblasts cultured on TCPs and the decellularized dermis treated using DME-DNase and SDS24-DNase for 7 days. Scale bar: 1 mm. (d) Adhered cell number of rat primary fibroblasts cultured on the decellularized dermis treated using DME-DNase and SDS24-DNase for 7 days. *n* = 4. DME, dimethyl ether; SDS, sodium dodecyl sulfate.

When cells were seeded on the subcutaneous tissue and epidermal sides of the decellularized dermis, no cells were observed in the SDS-decellularized dermis (Figure 4c,d). This could be due to the lack of cell attachment or cell death; however, in conjunction with the results of coculture with cell culture inserts (Figure 4a,b), we speculated that the residual SDS in the SDS-decellularized dermis lysed the cells. Cells adhering to the subcritical DME-decellularized dermis were different in shape from the TCPs. Spherical cells and cells extending in all directions were observed on TCPs. In contrast, cells on the subcutaneous tissue side more often extended in one direction, whereas cells on the epidermal side more often extended in all directions. There was no change in the shape of cells cocultured in cell culture inserts (Figure 4a), it can be inferred that these changes in cell shape were influenced by the shape of the surface to which the cells adhered. Thus, the results indicate that the subcritical DME-decellularized dermis may retain the structural features that control cell morphology and behavior. The number of cells observed in the DME-decellularized dermis was approximately half of the number of cells on the TCPs. It is a general property of the fibroblasts used in this study to proliferate to confluence on the TCPs. In contrast, the cell density of fibroblasts in the dermis was low (Figure 1a). Therefore, the number of cells in the decellularized dermis, which has the same structural characteristics as the actual dermis, would also be lower than that in the TCPs.

### In Vivo Evaluation of Decellularized Dermis

Subcutaneous transplantation of the decellularized dermis in rats revealed infiltration and distribution of cells in the gaps between collagen fibers in both subcritical-zed and SDS-decellularized dermis (Figure 5a). No significant inflammatory response was observed in either case. The number of cells infiltrating the subcritical DME-decellularized dermis was higher on the dermal side than on the epithelial side, and the central area was almost cell-free, even at 28 days (Figure 5b). The number of cells on the epithelial side and in the center of the SDS-decellularized dermis was comparable to that of the subcritical DME-decellularized dermis. The number of cells on the dermal side of the SDS-decellularized dermis was low on day 7 but increased by day 28. A possible reason for this could be that, as in the in vitro experiments, the residual SDS in the decellularized dermis affected the cell invasiveness of the SDS decellularized dermis. Despite differences in the area and number of infiltrated cells, the distribution of infiltrated cells in the interstitial spaces of the collagen fibers was similar to that in the untreated dermis (Figure 1a). Thus, the cells were recellularized in both the subcritical DME-decellularized and SDS-decellularized dermis.

**Figure 5:**
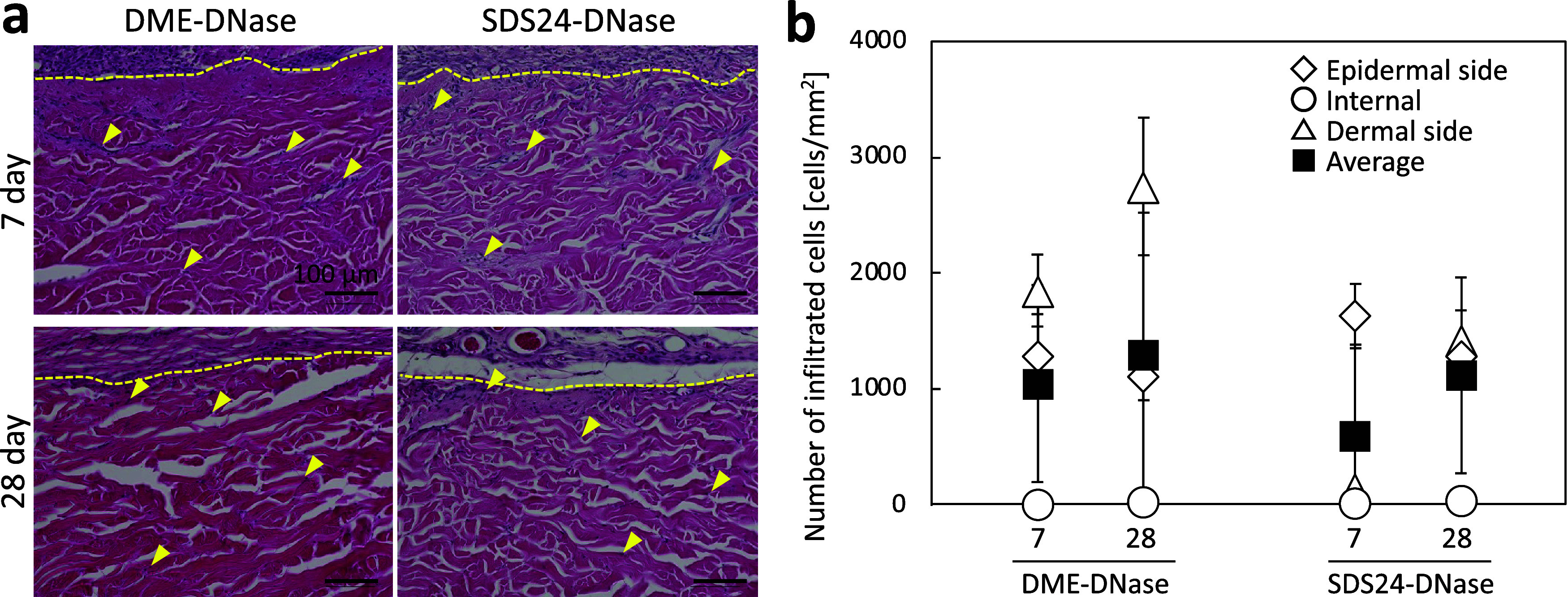
Cell infiltration into decellularized dermis in vivo. (a) Hematoxylin and eosin-stained image of the epithelial side of the transplanted decellularized dermis. The yellow dotted lines indicate the boundary between the decellularized dermis and the rat tissue. The yellow arrows indicate cell nuclei. (b) Number of cells infiltrating the transplanted decellularized dermis. The average number of cells in the epithelial, internal, and dermal side areas and their means are shown in the plots. DME, dimethyl ether; SDS, sodium dodecyl sulfate.

The ECM structure and mechanical properties of the subcritical DME-decellularized dermis were not significantly different from those of the SDS-decellularized dermis (Figure 2), and the cells responded well in vitro (Figure 4) and in vivo (Figure 5). Thus, the subcritical decellularized dermis is biocompatible and capable of tissue remodeling. In clinical applications of decellularized tissues prepared using surfactants, the potential adverse effects of residual surfactants remain a significant concern. Although not addressed in this study, additional steps such as prolonged washing are generally required to minimize surfactant residues. In contrast, dimethyl ether (DME), which readily evaporates under atmospheric pressure, does not require any special procedures for removal, thereby reducing the risk of residual toxicity.

Recently, there have been reports on the development of highly functional decellularized tissues with accelerated tissue regeneration by loading extracellular vesicles (EVs), such as exosomes, onto decellularized tissues.^25–27^ EVs are lipid bilayer vesicles that contain bioactive substances, such as mRNAs and proteins, and are important for intercellular communication. In previous reports, EVs were loaded onto prepared decellularized tissues; however, because biological tissues naturally contain cells before decellularization, it is possible that biological tissues originally contain EVs. Unlike surfactant treatment, the DME treatment used in this study disrupts only a portion of the lipid bilayer; therefore, it is expected that a large number of EVs will remain inside the decellularized tissue. To develop decellularized tissues with higher functionality, it is necessary to consider decellularization treatment methods that not only remove cellular components and retain ECM, but also consider new factors, such as EVs.

This study has several limitations that warrant consideration. First, the in vivo evaluation was restricted to short-term observations. Thus, long-term assessments of biocompatibility, immunogenicity, and degradation behavior were not conducted. Second, although the comparison between subcritical DME and surfactant decellularization methods yielded encouraging results for the DME approach, further comprehensive and quantitative analyses such as evaluation of EVs preservation and functional tissue remodeling are necessary to substantiate the clinical potential of this novel technique. Addressing these limitations in future studies will contribute to a more thorough understanding of the applicability and effectiveness of subcritical DME decellularization in tissue engineering.

## Conclusions

A subcritical DME decellularized dermis was prepared by removing DNA from the porcine dermis while retaining the structural and mechanical properties of the ECM by applying subcritical DME and DNase treatments in sequence. The subcritical DME decellularized dermis did not affect cell viability in vitro and exhibited cell adhesive properties. These cells also exhibited good in vivo invasiveness. Examination of the mechanism of decellularization revealed that surfactants decellularized cells mainly by washing away cellular components while dissolving the cell membrane, whereas DME caused minor damage to the cell membrane, which consists of a lipid bilayer and degraded cellular DNA by DNase. These results suggest that subcritical DME treatment is useful for preparing decellularized tissues with high functionality, such as residual EVs. Future studies should aim to evaluate long-term outcomes and functional performance, as well as to assess the preservation of EVs, in order to further clarify the potential and efficacy of subcritical DME decellularization for clinical applications.

## Experimental Section

### Preparation of Decellularized Dermis

Porcine skin was obtained from a slaughterhouse (Tokyo Shibaura Organ Co. Ltd., Tokyo, Japan) and trimmed to a diameter of 8 mm after removing the fat layer and hair. They were immersed in povidone iodine solution for 10 min and washed by saline and immersed in the gentamicin solution for 12 h at 4 °C for sterilization.

For the preparation of the decellularized dermis using subcritical DME, the samples trimmed to a diameter of 8 mm were placed in an extraction container (25-MLTH, Waters, MA) and perfused in a fixed direction for 5 h at 25 °C with liquefied DME (Air Can 420D, TAMIYA, Inc., Shizuoka, Japan) by applying a pressure of 0.7 MPa.^9–11^ A high pressure syringe pump (260D TELEDYNE ISCO, NE) was used to adjust the pressure during perfusion. To decompose DNA and remove cellular components, they were shaken moderately for 72 h at 4 °C in 50 mL of saline containing 50 mM magnesium chloride, 0.2 mg/mL of DNase I (Roche, Indianapolis, IN), and antibiotics. The samples then were washed with saline for 72 h at 4 °C. The treated dermises were stored in saline at 4 °C until used.

For the preparation of the decellularized dermis using surfactants, the samples trimmed to a diameter of 8 mm were immersed in 50 mL of 0.5% SDS solution and shaken for 5 or 24 h at approximately 25 °C. They were then shaken moderately for 72 h at 4 °C in 50 mL of saline containing 50 mM magnesium chloride, 0.2 mg/mL of DNase I, and antibiotics, and washed using saline for 72 h at 4 °C. The treated dermises with surfactant were stored in saline at 4 °C until used.

### Histology

The samples were fixed in 10% neutral buffered formalin at room temperature for 24 h. They were dehydrated with ethanol and xylene and embedded in paraffin. The paraffin block was sliced into 4-μm thick sections. Sections were treated to remove paraffin and stained with H&E and EVG. The stained sections were observed using a bright-field microscope (BZ-X710; KEYENCE, Osaka, Japan).

### Quantification of Residual Phospholipid

Each decellularized sample (8 mm in diameter) was homogenized with 1 mL of a solvent mixture of chloroform and methanol (5:1). The samples and solvent were filtered through a 1.0-μm membrane filter. The solvent was removed using a vacuum evaporator and the residue was dissolved in tetrahydrofuran. Phospholipid content was quantified by measuring absorbance using the phospholipid C test (Fujifilm/Wako Chemical, Osaka, Japan).

### Quantification of Residual DNA

Each decellularized sample was freeze-dried and weighed, then cut into small pieces and placed into individual tubes at 20 mg per sample. To each tube, 50 μL of proteinase-K solution (50 mg/mL; Wako Pure Chemicals, Osaka, Japan) and 500 μL of a buffer containing 50 mM Tris-HCl, 25 mM EDTA-2Na, 100 mM NaCl, and 1% SDS were added. The samples were incubated at 55 °C overnight for digestion. DNA was isolated using phenol/chloroform extraction and collected using ethanol precipitation. The obtained DNA solution was analyzed using PicoGreen. DNA content per sample was normalized to the dry weight of the corresponding samples.

### Mechanical Properties

The mechanical strengths of the untreated and treated dermis were evaluated. All the samples were cut into dumbbell-shaped pieces. The test pieces were 16 mm long and 4 mm wide. The thickness was measured using a Vernier caliper. Each sample was strained at the rate of 1 mm/s. The loads were measured using a force tester (MCT-2150, A&D Company, Limited, Tokyo, Japan). To obtain the stress–strain curves, the stress was calculated by normalizing the load by the initial cross-sectional area (N/mm^2^), and the strain was calculated by normalizing the displacement by the initial length of the samples. To account for initial slack in the tissue, the point at which the load began to increase continuously was defined as the zero-strain point. From the stress–strain curves, the initial elastic modulus (ε = 0–0.05) and the tangent elastic modulus (ε = 0.2–0.4) were calculated for each sample. The values obtained from each group were averaged (*n* = 3).

### Destruction of Cell Membranes and Removal of Nuclei in Cultured Cells

The L929 mouse fibroblast-like cell line, were maintained in Dulbecco’s modified Eagle’s medium (DMEM; Life Technologies Japan Ltd., Tokyo, Japan) supplemented with 10% fetal bovine serum (FBS, PAA Laboratories GmbH, Pasching, Austria), 100 U/mL penicillin, and 100 μg/mL streptomycin (Life Technologies Japan Ltd.) at 37 °C in a humidified atmosphere of 5% CO_2_ and subcultured at 1:8 split every 3 to 4 days. Glasses (7 mm × 7 mm) were placed in a 24-well plate. L929 cells were seeded at 1.25 × 10^5^ cells/sample. After 48 h, the cells adhering to the glass slides were treated with DME, EtOH, TritonX-100, or SDS. For DME treatment, the samples were placed in a pressure-resistant container containing 10 mL of DMEM. After adding 20 mL of liquefied DME at 25 °C and 0.7 MPa, the samples were immersed in liquefied DME for 1 h. For EtOH treatment, the samples were immersed in 0.2 mL of 100% EtOH for 1 min and washed with PBS. For SDS treatment, the samples were immersed in 0.02 mL of 0.02% SDS in PBS for 1 min and washed with PBS. For TritonX-100 treatment, the samples were immersed in 0.2 mL of 0.05% TritonX-100 in PBS for 1 min and washed with PBS. To evaluate cell membrane destruction, treated samples were stained with Calcein-AM and PI solutions, and observed under a fluorescence microscope (BZ-X710; KEYENCE). All fluorescence images were captured under identical imaging conditions. To evaluate the structure of cell membranes, the treated samples were fixed in 2.5% glutaraldehyde in PBS at room temperature for 24 h, they were dehydrated with ethanol, immersed overnight in *tert*-butyl alcohol, and freeze-dried. The dried samples were sputter-coated with gold and observed using SEM with a model S-3400N apparatus (Hitachi High-Technologies Corporation, Tokyo, Japan).

The cells treated using DME, EtOH, TritonX-100, and SDS were immersed for 24, 48, 72 h in saline containing 50 mM magnesium chloride, 0.2 mg/mL DNase I, and antibiotics. To evaluate the cell nucleus removal, the treated samples were stained with Calcein-AM and PI solutions and observed using phase contrast and fluorescence microscopy. Results are expressed as mean ± standard deviation. Statistical significance was determined using two-way ANOVA. Statistical significance was set at *p* < 0.05. significant.

### Primary Culture of Rat Fibroblasts

Wistar rats (male, 6 weeks old, Tokyo Experimental Animals, Tokyo, Japan) were used in this study. The skin of the abdomen was harvested after hair removal and disinfection. The samples were washed with PBS and placed in the cell culture dish. DMEM supplemented with 20% FBS (10 mL), 100 U/mL of penicillin, and 100 μg/mL of streptomycin were added. They were incubated for 7 days at 37 °C in a humidified atmosphere of 5% CO_2_. They were subcultured in 4 × 10^5^ cells/100 mm culture dishes every 3–4 days. Second-passage cells were used in the experiments.

### Culturing of Rat Fibroblasts with the Decellularized Dermis

Rat fibroblasts were seeded into a 12-well plate at a density of 5 × 10^4^ cells/well in 2 mL of culture medium. Six hours after cell seeding, cell culture inserts were placed in each well. Half of each decellularized dermis sample (8 mm in diameter) was finely chopped and added to the corresponding insert. The cells were then cultured for 7 days. The cells were evaluated using a phase-contrast microscope (BZ-X710; KEYENCE) and the number of adhered cells was counted. Results are expressed as mean ± standard deviation. Statistical significance was determined using a two-way ANOVA. Statistical significance was set at *p* < 0.05.

### Culturing of Rat Fibroblasts on the Decellularized Dermis

Decellularized dermis samples were individually placed on the 96-well plate and immersed on the culture medium for 1 h. After removing the medium, the rat fibroblasts were seeded on the decellularized dermis of the subcutaneous tissue or epidermis at 5 × 10^4^ cells/sample. Seven days after seeding, the cells were stained with Calcein-AM and PI solutions, and observed using fluorescence microscopy. All fluorescence images were captured under identical imaging conditions. The number of cells adhering to the samples was counted using a Hybrid Cell Count Application (BZ-H3C; KEYENCE).

### Subcutaneous Implantation

All experiments involving rats were conducted using protocols approved by the Institutional Animal Care and Use Committee of Shibaura Institute of Technology (19013). Wistar rats (male, 6-week old, Tokyo Experimental Animals) were used in this study. The samples were subcutaneously implanted. Subcutaneous pockets were opened on the backs of rats under deep general anesthesia. The samples were inserted into the pockets with the epidermal side of the decellularized dermis facing outward and sutured. Seven and 28 days after implantation, the samples were harvested and evaluated using H&E staining. The cell nuclei observed on the epidermal side, central part, and subcutaneous tissue side of the transplanted sample were counted visually. The area of the transplanted sample in each image was calculated, and the number of infiltrating cells per area was calculated.

## Abbreviations

DME: dimethyl ether
ECM: extracellular matrix
EV: extracellular vesicle
EVG: Elastica van Gieson
SDS: sodium dodecyl sulfate
SEM: scanning electron microscopy
